# A Case of Neuroschistosomiasis Presenting as Transverse Myelitis: The Importance of History Taking

**DOI:** 10.7759/cureus.11445

**Published:** 2020-11-11

**Authors:** Ahmad S Matarneh, Wafa Abdullah, Adeel A Khan, Amna Sadiq, Khalid Farooqui

**Affiliations:** 1 Internal Medicine, Hamad Medical Corporation, Doha, QAT; 2 Radiology, Hamad Medical Corporation, Doha, QAT

**Keywords:** neuroschistosomiasis, schistosomiasis, transverse myelitis, helminthic

## Abstract

Neuroschistosomiasis is a rare manifestation of Schistosoma infection and can either manifest as cerebritis or with spinal cord involvement. We present a case of low back pain and lower limb weakness, which was initially managed as idiopathic transverse myelitis and later on found to have neuroschistosomiasis.

A 23-year-old Sudanese gentleman presented with a one-week history of low back pain, lower limb weakness, and urinary retention. An urgent MRI of the spine with contrast showed features suggestive of transverse myelitis. The patient was treated with intravenous methylprednisolone for five days, which showed significant improvement in his symptoms. One week later, the patient developed the same symptoms again. An urgent MRI spine showed an interval progression of MRI findings. Repeat history taking revealed a history of swimming many times in the river Nile. Serology was sent for Schistosoma and came positive with titer 1:1280. He was treated as neuroschistosomiasis with intravenous steroids for three days, followed by praziquantel for five days along with the steroids, after which he showed significant improvement in his lower limb weakness.

Spinal neuroschistosomiasis is one of the very rare complications of Schistosoma infection that should be kept in mind when dealing with unexplained myelopathy with a history of travel or origin from an endemic area. If not treated promptly, it can result in severe irreversible complications.

## Introduction

Schistosomiasis is a helminthic disease and is considered a common parasitic infection in the Middle East [1]. Infection usually occurs after contact with the intermediate host, which are snails in freshwater, containing the eggs and parasite. Snails then release cercariae, the infectious form of the parasite, into the water that can affect hosts after penetrating the skin [2]. Neuroschistosomiasis is a rare manifestation of the disease. It can either involve the brain where it can cause cerebritis or involve the spinal cord [3]. In this case, we present a rare clinical scenario in which the patient presented with lower limb weakness and was found to have transverse myelitis secondary to neuroschistosomiasis and showed significant improvement after treatment.

## Case presentation

A previously healthy 23-year-old Sudanese gentleman presented to the hospital with a one-week history of low back pain, dull in character, occurring mainly at night associated with difficulty in urination and weakness in both legs for three days. Lower limb weakness was symmetrical and progressed over one day till he became unable to move his legs. He denied weakness in arms or any disturbance in sensations. There was no recent history of fever, sore throat, cough, night sweats, weight loss, or trauma to the back. No personal or family history of similar conditions was reported. The patient was a non-smoker, and there was no history of alcohol abuse. He was living in Qatar, and his last travel to Sudan was eight months ago. He worked as a guard and mentioned a history of lifting heavy objects at work.

On examination, the patient had normal vital signs. Neurological examination was remarkable for hypertonia in both lower limbs. Power was 4/5 in proximal muscles and 1/5 in distal muscles. He had exaggerated knee jerk reflexes bilaterally but normal ankle jerk reflexes. Plantar reflexes were flexor bilaterally. The sensory and cerebellar examination was normal. Back examination revealed tenderness over the sacral area. Cardiac, respiratory, and abdominal examinations were normal. Complete blood count, urea, creatinine, electrolytes, C-reactive protein, procalcitonin, vitamin B12, folic acid, and thyroid function tests were normal (Table 1).

**Table 1:** Basic laboratory investigations WBC: white blood cell, Hgb: hemoglobin, AST: aspartate aminotransferase, ALT: alanine aminotransferase, ALP: alkaline phosphatase, CRP: C-reactive protein, ANA: anti-nuclear antibody

Test	Result	Reference Range
WBC count	4.9×10^6 /ul	4.5 – 5.5 ×10^6 /ul
Hgb	14.3 gm/dl	13 - 17 gm/dl
Urea	2.90 mmol/L	2.8 – 8.1 mmol/L
Creatinine	78.0 umol/L	62 – 106 umol/L
Sodium	139.0 mmol/L	136 – 145 mmol/L
Potassium	3.90 mmol/L	3.5 – 5.1 mmol/L
Calcium	2.34 mmol/L	2.15 – 2.50 mmol/L
AST	49.0 U/L	0 – 40 U/L
ALT	21.0 U/L	0 – 41 U/L
ALP	65.0 U/L	40 – 129 U/L
CRP	0.5 mg/dl	0.0 – 5.0 mg/dl
Lactic acid	1.0 mmol/L	0.5 – 2.2 mmol/L
Procalcitonin	0.31 ng/ml	< 0.5 ng/mL
Vitamin B12	196.0 pmol/L	145 – 596 pmol/L
ANA	Negative	
C3	0.82 gm/dl	0.8 – 1.8 gm/dl
C4	0.14 gm/dl	0.1 – 0.40 gm/dl

**Figure 1:**
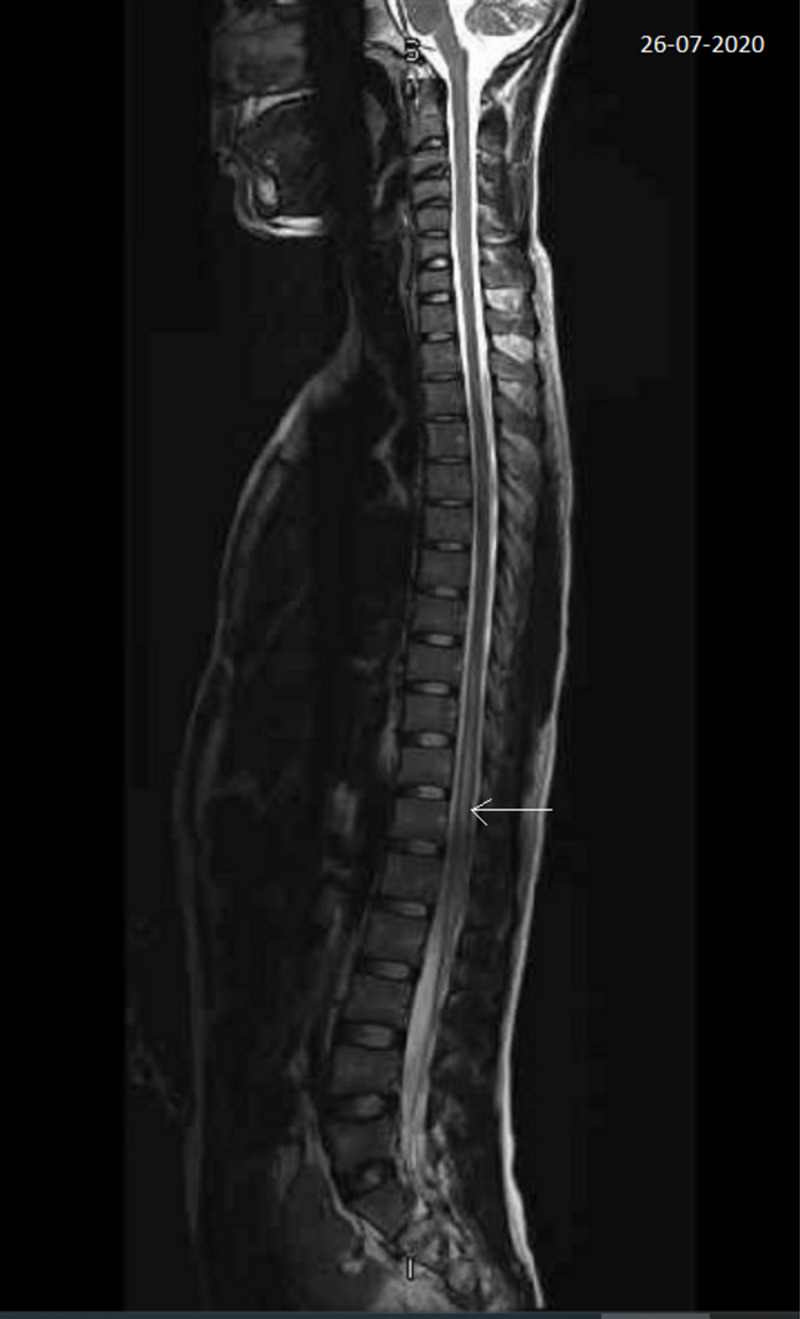
T2 bright signal of the long segment of lower dorsal spinal cord and conus medullaris (mainly central and bilateral intramedullary) with focal T12/L1 levels faint postcontrast enhancement suggestive of transverse myelitis

Autoimmune profile, blood culture, serology for brucella, syphilis, hepatitis B, Hepatitis C, and HIV were negative. The chest X-ray was normal. An urgent MRI of the spine with contrast showed a T2 bright signal of a long segment of the lower dorsal spinal cord and conus medullaris (mainly central and bilateral intramedullary) with focal T12/L1 levels faint postcontrast enhancement suggestive of transverse myelitis (Figure 1).

Cerebrospinal fluid (CSF) analysis showed 201 white blood cells (WBCs) with 95% lymphocytes. Glucose was 4.52 mmol/L and proteins 0.45 gm/L (Table 2). The patient was diagnosed with idiopathic transverse myelitis and started on intravenous (IV) methylprednisolone for five days, which resulted in significant improvement in his symptoms. He was then transferred to a rehabilitation facility.

**Table 2:** CSF analysis CSF: cerebrospinal fluid, AFB: acid-fast bacillus

Test	Result	Reference Range
Colour	Colourless	--
White blood cells	201 /uL	0 – 5 /uL
Red blood cells	15 /uL	0 – 2 /ul
Lymphocytes	95%	40 – 80 %
Monocytes	2%	15 – 45 %
Glucose	4.52 mmol/L	2.22 – 03.89 mmol/L
Proteins	0.45 gm/L	0.15 – 0.45 gm/L
AFB smear	Negative	
Oligoclonal bands	Negative	
Viral panel	Negative	
Culture	Negative	

**Figure 2:**
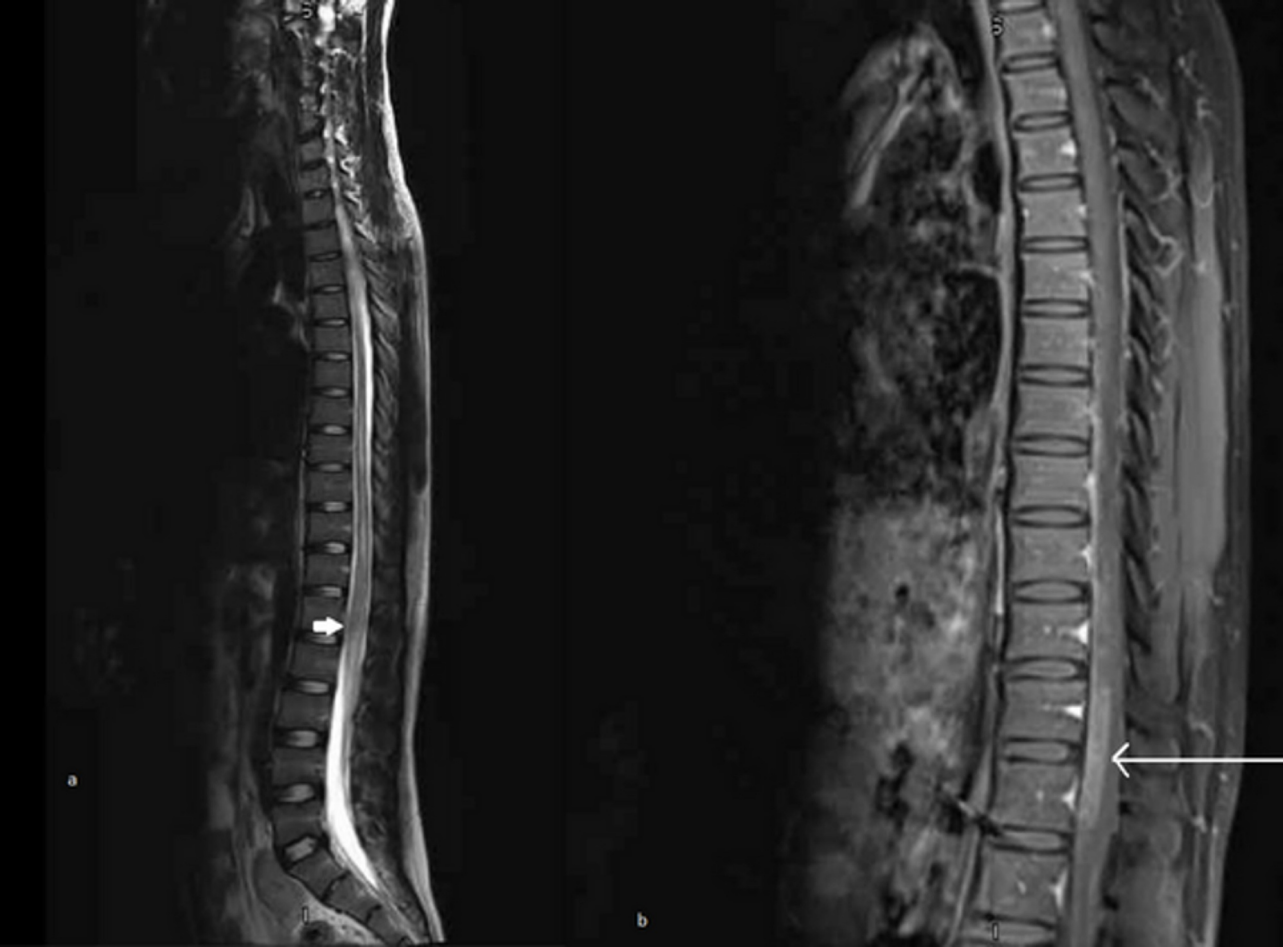
MRI thoracolumbar spine with contrast showing interval increase in intramedullary T2 enhanced signals. There is also a prominent enhancement of cauda equina root. Oedema of conus (arrow in image a). New patchy enhancement noted in the spinal cord at T10-T11 level (image b)

One week later, the patient developed the same symptoms again. An urgent MRI spine showed the progression of his disease (Figures 2a, 2b). A repeat CSF examination showed 54 WBCs with 91% lymphocytes (Table 3).

**Table 3:** CSF analysis second admission CSF: cerebrospinal fluid, AFB: acid-fast bacillus

Test	Result	Reference Range
Colour	Colourless	--
White blood cells	54 /uL	0 – 5 /uL
Red blood cells	3 /uL	0 – 2 /ul
Lymphocytes	91%	40 – 80 %
Monocytes	5%	15 – 45 %
Glucose	2.99 mmol/L	2.22 – 03.89 mmol/L
Proteins	1.36 gm/L	0.15 – 0.45 gm/L
AFB smear	Negative	
Oligoclonal bands	Negative	
Viral panel	Negative	
Culture	Negative	

CT chest showed tree in bud appearance, raising suspicion of pulmonary tuberculosis. However, acid-fast bacillus (AFB) smear and polymerase chain reaction (PCR) from sputum and bronchoalveolar lavage were negative. CT abdomen and pelvis were unremarkable. When retaking history, he mentioned swimming frequently in the river Nile. Given the high incidence of Schistosoma in swimmers, serology for Schistosoma was sent and came positive with titer 1:1280. Hence, he was diagnosed with neuroschistosomiasis and started on intravenous steroids for three days, followed by praziquantel for five days along with the steroids, after which he showed significant improvement in his lower limb weakness. He was transferred to the rehabilitation center and kept on oral steroids for six weeks with good tolerance to medications; he continued to improve.

## Discussion

Schistosomiasis is a rare helminthic disease caused by Schistosoma flukes that can affect multiple organs, including the liver, intestines, lungs, urinary bladder, brain, and spinal cord. There are several known types of Schistosoma, namely, S. haematobium, S. intercalatum, S. japonicum, S. mansoni, and S. mekongi [4]. It is regarded as the second most common tropical disease [5]. Neuroschistosmiasis is usually a rare presentation resulting from the embolization of the organism’s eggs through the vasculature until it lands in the central nervous system. It is divided into cerebral Schistosoma, which occurs when S. japonicum reaches the brain causing encephalitis causing symptoms of headache, seizures, altered mentation or the spinal schistosomiasis resulting from S. mansoni (and less commonly S. haematobium) where it can cause myelitis with symptoms of weakness, back pain and urine retention (Poster: Elsbernd P, Lago K, Calvano T, Sladky J. Complete Neurologic Recovery after Acute Cauda Equina Syndrome due to Neuroschistosomiasis. AAN 70th Annual Meeting; April 26, 2018). After the eggs of the organism reach the central nervous system, they mature into adult forms. Schistosoma eggs usually induce a local eosinophilic inflammation resulting from the release of proteolytic enzymes. The resultant inflammation causes damage and granuloma formation, and eventually fibrosis and demyelination of the surrounding structures [6].

A high index of clinical suspicion based on epidemiological stratification is required to make the diagnosis of neuroschistosomiasis. MRI is the imaging modality of choice to diagnose central nervous system (CNS) involvement and it can detect changes consistent with acute myelitis and spinal cord compression secondary to granuloma [7,8]. A definite diagnosis is made by tissue biopsy. Serology is a sensitive test for the diagnosis, but with high false-positive rates, they are sometimes considered un-reliable, however, positive test results with high titers (>1:160) are considered significant [6,9].

After establishing the diagnosis, rapid treatment should always be sought as it has a significant benefit in improving the outcome. Steroids are usually started before praziquantel to decrease the inflammation that might result from the cytotoxic effect of praziquantel on the organism [10]. Praziquantel acts by increasing the membrane permeability to calcium, thereby causing tetanic contractions and paralyzing the organism [11]. Moreover, it acts only on the mature adult worms and not the larval form rendering it ineffective in the early stages of infection [12]. Other lines of treatment include artemisinin, of limited efficacy, and oxamniquine, which is only effective on S. mansoni [13].

Our patient was initially diagnosed with transverse myelitis and received IV steroids for five successive days with significant improvement. However, his symptoms worsened shortly after stopping the steroids. On repeated history taking, the patient reported a history of swimming in freshwater in Sudan, which, combined with high titers of antibodies, raised suspicion of neuroschistosomiasis as a cause.

## Conclusions

Spinal neuroschistosomiasis is one of the very rare presentations of Schistosoma infection that should be kept in mind when dealing with unexplained myelopathy with a history of travel or origin from an endemic area. If not treated promptly, it can result in severe irreversible complications. Treatment with steroids before initiating praziquantel can help in decreasing the risk of disease progression, improving morbidity and overall outcome.
